# *ALDH1L2* Knockout in U251 Glioblastoma Cells Reduces Tumor Sphere Formation by Increasing Oxidative Stress and Suppressing Methionine Dependency

**DOI:** 10.3390/nu14091887

**Published:** 2022-04-30

**Authors:** Maëlle Quéré, Jean-Marc Alberto, Franck Broly, Sébastien Hergalant, Christo Christov, Guillaume Gauchotte, Jean-Louis Guéant, Farès Namour, Shyue-Fang Battaglia-Hsu

**Affiliations:** 1 INSERM U1256, NGERE—Nutrition, Genetics, and Environmental Risk Exposure, University of Lorraine, 54500 Nancy, France; maelle.quere@univ-lorraine.fr (M.Q.); jean-marc.alberto@univ-lorraine.fr (J.-M.A.); sebastien.hergalant@inserm.fr (S.H.); christo.christov@univ-lorraine.fr (C.C.); guillaume.gauchotte@univ-lorraine.fr (G.G.); jean-louis.gueant@univ-lorraine.fr (J.-L.G.); 2 Service de Génopathies, Centre de Biologie Pathologie Génétique, Centre Hospitalier Régional et Universitaire de Lille, CEDEX, 59037 Lille, France; franck.broly@chru-lille.fr; 3 Department of Biopathology, CHRU Nancy, Rue du Morvan, 54511 Vandoeuvre-les-Nancy, France; 4 Department of Molecular Medicine, Division of Biochemistry, Molecular Biology, Nutrition, and Metabolism, University Hospital of Nancy, 54505 Vandoeuvre-les-Nancy, France

**Keywords:** tumor sphere, glioblastoma, methionine, ROS, *ALDH1L2*

## Abstract

Previously, the in vitro growth of cancer stem cells in the form of tumor spheres from five different brain cancer cell lines was found to be methionine-dependent. As this earlier work indicated that *ALDH1L2*, a folate-dependent mitochondria aldehyde dehydrogenase gene, is upregulated in glioblastoma stem cells, we invalidated this gene using CRISPR-cas 9 technique in this present work. We reported here that this invalidation was effective in U251 glioblastoma cells, and no cas9 off target site could be detected by genome sequencing of the two independent knockout targeting either exon I or exon III. The knockout of *ALDH1L2* gene in U251 cells rendered the growth of the cancer stem cells of U251 methionine independent. In addition, a much higher ROS (reactive oxygen radicals) level can be detected in the knockout cells compared to the wild type cells. Our evidence here linked the excessive ROS level of the knockout cells to reduced total cellular NADPH. Our evidence suggested also that the cause of the slower growth of the knockout turmor sphere may be related to its partial differentiation.

## 1. Background

Methionine is the precursor of the universal methyl donor, S-adenosyl-methionine, for most methylation reactions taking place inside cells. Methionine cycle and folate cycle together constitute the key components of the one carbon (1C) metabolism used by cells to feed 1C units to various intermediate metabolites for biological processes [1]. 1C metabolism takes place in both cytosol and mitochondria, and these two compartments are linked through formate produced by the mitochondria folate cycle. Once produced, mitochondrial formate moves freely into cytoplasm for conversion to downstream cytosolic folates for de novo synthesis of either methionine or nucleic acids [2]. One major byproduct of folate cycle is NADPH [3], a crucial electron donor powering cellular level anabolic and redox reactions.

Metabolic reprogramming in pluripotent stem cells and cancer stem cells (CSC) share many similarities. Among them, the methionine-dependent growth [4,5] and the hypersensitivity to reactive oxygen species (ROS), the latter due to reprogramming of NADPH metabolic pathway that renders these cells highly sensitive to oxidative stress [6]. In fact, ROS level plays a key role in the control of stem cell fate as a high ROS level can trigger senescence and death [7], whereas a lower ROS level can help to preserve stemness [8] and cause tumor radioresistance [9]. It is worth noting that the ROS signaling in CSC is especially dynamic, shaped not only by the constantly changing microenvironment but also by the metabolic plasticity of the CSC. Such plasticity is likely a key adaptive mechanism of these cells to ensure their survival. Our previous work suggested that 1C metabolism takes part in this adaptive response to maintain the CSC population in U251 glioblastoma cells.

The ROS scavenging aldehyde dehydrogenases (ALDH) have been used as markers for stem cells [10]. These enzymes have multiple functional roles in CSCs, including also aldehyde detoxification and retinoic acid signaling [11]. They are implicated in therapeutic resistance to chemotherapy [12,13], and pharmacological inhibitors of ALDH activity can abolish stem cell characteristics [14]. ALDH1L2, 10-formyltetrahydrofolate dehydrogenase and a mitochondrial 1C cycle folate enzyme, can reduce ROS production and promote metastasis in certain contexts [15]. In the neuro-ichthyotic syndromes, the only known human diseases associated with *ALDH1L2* mutations, the loss of *ALDH1L2* activity causes diminished NADPH production, elevated ROS, increased oxidative stress, and impaired mitochondrial function [16]. NADPH combats oxidative stress and acts either by generating reduced glutathione (GSH) or as a direct antioxidant [17]. Thus, a loss of function of ALDH(1L2) can exacerbate oxidative stress and mitochondrial dysfunction.

1C metabolism plays a pivotal role in stem cell proliferation as methionine cycle is capable of providing both methionine and SAM for the proliferation of these cells, and folate cycle can supply NADPH for antioxidative protection against ROS. In earlier work, the methionine growth dependency of the CSC of U251 glioblastoma was attributed to reduced 5-methyl-tetrahydrofolate [18]. Comparative transcriptomics between the differentiated and CSC U251 cells revealed potential activation of the mitochondrial folate cycle in CSC as several key mitochondria folate enzymes, including ALDH1L2, are upregulated while several cytosolic folate enzymes are downregulated in these CSC cells.

The objective of this current work was thus to determine the role played by the up-regulated ALDH1L2 in CSC obtained in vitro in the form of tumor sphere (TS). To achieve this, we invalidated ALDH1L2 using a CRISPR Cas9 system. As ALDH1L2 converts mitochondrial 1C donor 10-formylTHF to CO_2_ and THF, we expected that its knockout will help to preserve the 1C units, and hence render the growth of CSC methionine-independent. In addition, because the cofactor needed for this ALDH1L2-dependent oxidation of mitochondrial 10-formylTHF is NADP^+^, we expected that this knockout will reduce the cellular content of NADPH. As NADPH is a major reducing power for anti-oxidative defense, we expected to see an increased ROS level in the CSC knockout cells.

## 2. Methods

### 2.1. Generation of ALDH1L2 CRSPR Knockouts (KO)

To create ALDH1L2 KO cells, U251 were transfected with the lentivirus particles. This was followed by cloning. For each isolated clone, ALDH1L2 gene deletion was evaluated by targeted Sanger sequencing as well as genome sequencing (Supplemental Methods).

### 2.2. Whole Genome Sequencing (WGS) 

Whole genome sequencing (WGS) and primary analysis of the results were performed by BGI (Beijing Genomics Institute, Shen-Zhen, China). The secondary and tertiary analyses (alignment of reads on the reference human genomic sequence, detection_annotation_visualization of variants) were carried out (Supplemental Methods).

### 2.3. Metabolites Analysis

Metabolites analysis including Methionine, Homocysteine, SAM, SAH, total Cysteine, Cystathionine, total Glutathione, Choline, Betaine, Glycine, Serine, methyl-THF and formyl-THF were realized by LCMS (LCMS 8045, Shimadzu, Kyoto, Japan) on a Kinetex column (Kinetex 00D-4462-EO). These measurements were performed as detailed in the Supplemental Methods section.

### 2.4. Statistical Analysis

Data generated by cell experiments were analyzed with ANOVA and Student’s *t*-test and were presented as mean ± standard error of the mean (s.e.m). All the reported *p*-values are two-sided, and *p*-values < 0.05 are considered statistically significant.

## 3. Results

### 3.1. CRISPR-Cas9 Targeting Either Exon 1 or Exon 3 of ALDH1L2 Abolishes ALDH1L2 Expression

In a previous study comparing stem-like verse differentiated glioblastoma cells established under in vitro culture conditions as tumor sphere (TS), we observed differential folate enzyme expressions, with tumor spheres showing higher mitochondria folate activity [18]. The higher expression of the mitochondrial ALDH1L2 in TS was particularly noted. In the present study, we hypothesized that ALDH1L2 is crucial for the formation of tumor spheres in glioblastoma.

**Figure 1:**
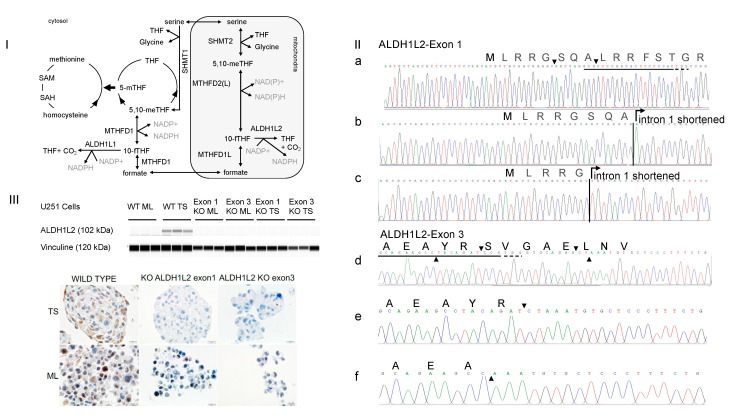
*ALDH1L2* KO U251 glioblastoma cells were generated using the CRISPER-Cas9 system (**I**) One carbon metabolism features linked mitochondrial and cytosolic pathways; folate and méthionine cycles are intertwinned in the cytosol. THF: TetraHydroFolate, 5,10-meTHF: 5,10 methylene THF, 10-fTHF: 10-formylTHF, 5-mTHF: 5 methyl THF, SAM: S-adenosyl methionine, SAH: S-adenosyl homocysteine. (**II**) Genomic Sanger sequencing of individual deletion clones shows bi-allelic disruption of targeted exons 1 and 3. For exon 1, the new sequences diverge from the wild type sequence by deletions of 42 and 62 bp. Wild type sequence is shown in (**a**) with arrows indicating the breaking points. Sequencing of the 42 deletion including 24 bp of exon 1 and 18 bp of intron 1 is shown in (**b**) and sequencing of the 62 bp deletion including 33 bp of exon 1 and 29 bp of intron 1 in (**c**). The consequences of these deletions on protein synthesis are unpredictable since they carry away the 5′ end of intron 1. For exon 3, the divergence is due to deletions of 14 and 23 bp. Wild type sequence is shown in (**d**) with top and bottom arrows indicating the 14 and 23 bp deletion boundaries inside exon 3. Sequencing of the 14 and 23 deleted exon 3 is shown in (**e**,**f**) with arrows indicating the breaking points. Both deletions were mapped and confirmed to yield the same truncated protein with a stop codon generated at codon 113 of the newly synthesized proteins. Sequences targeted by the sgRNA are underlined in solid lines and PAM sequences in dashed line. Amino acid sequences are depicted in single letter code above nucleotide sequences. (**III**) WES^TM^ analysis of ALDH1L2 protein in U251cells in the upper panel shows that ALDH1L2 is detected in wt TS but not in the deletion clones. It is of note that ALDH1L2 is weaky expressed in the wt ML. Immunohistolological staining of the ALDH1L2 in wt and knockout cells in the lower panel. Abbreviations, wt: Wild Type; ML: MonoLayer Cells; TS: Tumor Spheres; KO: knockout.

To test, we produced two lines of ALDH1L2 KO U251 glioblastoma cells using the CRISPR-Cas 9 targeting exon 1 and exon 3 of the human *ALDH1L2*, respectively. Sanger sequencing of the *ALDH1L2* KO clones showed bi-allelic disruption of the targeted exon 1 and 3. For exon 1, we identified two INDELs. In Figure 1II, their Cas 9 break points are indicated by “▼” on the wild type sequence. The mutant sequences diverge from that of the wild type by a deletion of 42 bp in one and 62 bp in the other clone. The 42 bp deletion includes the 24 bp at the end of exon 1 and the 18 bp at the beginning of the intron 1 (IIb). The 62 bp deletion includes the 33 bp at the end exon 1 and 29 bp at the beginning of intron 1 (IIc). The consequences of these deletions on the protein synthesis are unpredictable since they include the 5′ end of intron 1. For exon 3, two clones were also identified, carrying a 14 pb and a 23 bp deletion, respectively. In Figure 1IId, the positions of the two deletions are marked by “▲” and “▼” on the wild type sequence. The final sequences of the exon 3 deletion mutants are shown in **e** and **f**, with “▼” and “▲” indicating the Cas 9 breaking points. Both exon 3 deletions were mapped and expected to yield the same truncated protein consequent to a stop codon generated at position 113. We further confirmed the relative ALDH1L2 expressions in the wild type and mutant clones under differentiated monolayer and non-adherent TS culture conditions using the Simple Western WES^TM^ protein analysis platform as well as immunohistological staining (Figure 1III).

To determine the off-target sites of the selected CRISPR-Cas9 *ALDH1L2* knockout cells, we performed whole genome sequencing. After mapping sequence reads to the reference genome, we used several software programs to examine the patterns of sequence alignment both near the on-target site and other sites in the genome to determine the possible presence of cas9 off-targets. Analyses of the reads spanning the cleavage site bound by CRIPSR-Cas9 in *ALDH1L2* exons 1 and 3 revealed the same indels identified by the Sanger sequencing. Nucleotide variations were also observed in several other regions. Closer inspection of the reads alignment there revealed that they are all located in regions of poor sequence quality, as observed as well in the wild type cells. Thus, the staggered alignment (see Supplement Figure S1) at these other sites is not due to the “off-target” effect of the CRIPSR-cas9 system, but rather to incorrect base callings.

### 3.2. Tumor Sphere (TS) Growth of the ALDH1L2 KO Cells Are Slower and Less Methionine-Dependent

**Figure 2:**
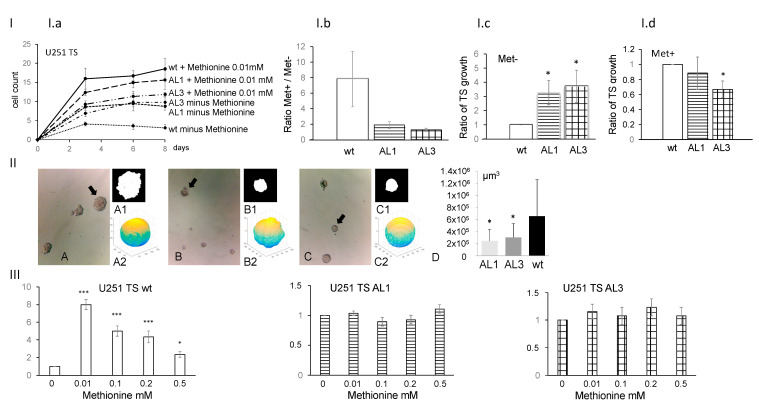
Tumor spheres (TS) derived from U251 *ALDH1L2* KO cells display not only decreased growth but also methionine-independent growth. TS growth in methionine free (MET−) vs. methionine (0.01 mM)-containing (MET+) media were compared between KO vs. wt cells. In (**Ia**), in MET−, TS of the KO cells grew better than that of the wt. In (**Ib**), the TS growth ratio was calculated as TS growth in MET+) divided by that in MET−. The higher the ratio, the more the methionine growth dependency. In (**Ic**), TS growth in MET− was normalized to that of the wt TS in MET−. In (**Id**), TS growth in MET+ was normalized to that of the wt TS MET+. In (**II**), analyses of the computer-assisted reconstruction of sphere volumes revealed that *ALDH1L2* KO has smaller “tumor sphere volume”. A, A1, A2 = wt cells. B, B1, B2 = AL1 cells. C, C1, C2 = AL3 cells. A1, B1, C1 are results of segmentation. A2, B2, C2 are results of 3D reconstructions: axes values are in pixels, and images are not to scale, so size differences can be appreciated visually in A1, B1, C1 but are best demonstrated by measured volumes: A = 1,857,000 µm^3^, B = 258,000 µm^3^, C = 206,000 µm^3^. D: Spheroid volumes versus wt (µm^3^), mean ± SD, * *p* < 0.003, *** *p* < 0.001, Student *t*-test. In (**III**), *ALDH1L2* KO suppressed U251 TS growth methionine dependency. For each cell type, mean ± SE of three independent experiments (each done in quadruplicate) is shown (three biological replicates each include four technical replicates). wt: wild type, AL1: U251 cells *ALDH1L2* exon 1 KO, AL3: U251 cells *ALDH1L2* exon 3 KO.

Armed with the *ALDH1L2* knockout clones, we examined the growth of TS derived from these cells either with or without exogenous methionine. We confirmed here that wild type U251 TS growth indeed requires methionine; however, this dependency is abolished in KO cells for both exon 1 (AL1) and exon 3 (AL3) KO cells (Figure 2III). As 0.01 mM appeared to be the optimal methionine concentration for wild type U251 TS growth, we compared the third passage of TS growth during an 8-day period under the condition of either methionine-free (MET−) or with 0.01 mM methionine (MET+) (Figure 2Ia). Specifically, on the 8th day, the impact of methionine on TS growth was compared. With MET− (Figure 2Ic), the total TS numbers obtained were 3.09 ± 0.89 (WT as 100%) vs. 8.76 ± 1.03 (AL1, 284%) and 9.79 ± 1.37 (AL3, 317%) (*p* < 0.05 for AL1 and *p* < 0.05 for AL3, respectively). With MET+, the total TS numbers obtained were 18.55 ± 2.68 (WT as 100%) vs. 15.63 ± 2.41 (AL1, 84%) and 11.85 ± 0.52 (AL3, 64%) (*p* = 0.46 and 0.07, respectively) (Figure 2Id MET+). We also compared the ratio of TS number (MET+)/TS number (MET−) for each cell line (Figure 2Ib). For wild type, this MET+/MET− ratios were 7.83 ± 3.59, while, for the two knockout cells, they were 1.89 ± 0.45, and 1.27 ± 0.21, respectively (*n* = 3, *p* < 0.05). These analyses all confirmed that wild type TS cells are much more methionine-dependent than either of the knockout cells. In addition to the TS number, we noted that the TS formed from the *ALDH1L2* KO cells were generally smaller than those observed from the wild type cells (Figure 2II; 194,830 µm^3^ and 229,430 µm^3^ vs. 610,190 µm^3^, *n* = 27 and *n* = 28 vs. n = 32 for AL1, AL3 and U251wt, respectively; *p* < 0.03).

### 3.3. ALDHL2 Knockout U251 Cells Have Reduced NADPH but Higher Cytosolic Methyl Donors in Tumor Spheres

**Figure 3:**
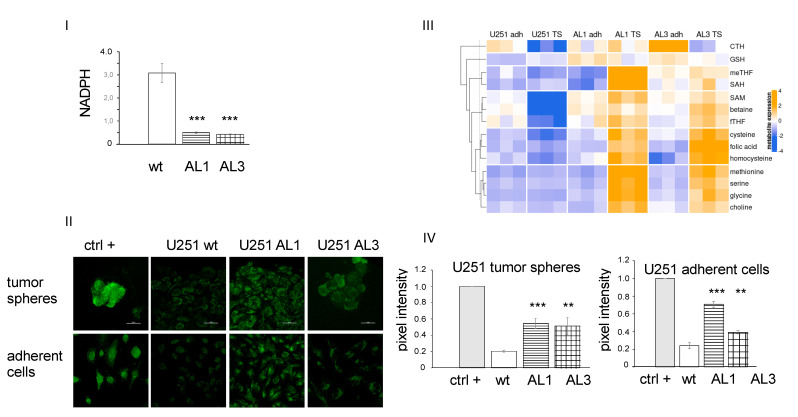
ALDH1L2 knockout U251 cells have lower NADPH level (**I**), no difference in GSH ratio (reduced GSH/total GSH) (**II**), and higher one carbon metabolites, including methionine, 5, methyl-tetrahydrofolate and formyl-tetrahydrofolate (**III**), as well as larger ROS production (**IV**). Tumor spheres were collected at passage 3 and adherent cells at 80% confluency. AL1 and AL3 were compared to U251 wild type, *n* = 6, ** *p* < 0.01, *** *p* < 0.001, AL1 = ALDH1L2 exon1 KO, AL3 = ALDH1L2 exon 3 KO.

In mitochondria, a number of substrates such as serine provide 1C units to produce mitochondrial 10-formyl-THF for downstream processes to either be oxidized to CO_2_ with simultaneous reduction of NADP+ to NADPH, or be transformed into formate, which, in turn, is integrated into a cytosolic folate cycle (see Figure 1I). These competing pathways are carried out by two distinct mitochondrial enzymes ALDH1L2 and MTHFD1L, respectively. This bifurcation is a key mechanism determining the fate of 1C units. In tumor spheres of the *ALDH1L2* knockout cells, this bifurcation is expected to tilt towards the production of mitochondria formate, leading to reduced mitochondria NADPH production while simultaneously fueling the generation of cytosolic 10-formyl THF to supply the cytosolic methionine cycle. These predictions were confirmed for TS cells (Figure 3). Relative to wild type TS, *ALDH1L2* knockout TS have significantly less total cellular NADPH (347.7 vs. 84.5 and 74.1 umol/g protein, respectively, for wt and AL1 and AL3 KO; *n* = 3, *p* < 0.01). As a result, the relative differences in the normalized TS to adherent NADPH ratio are greater than those not normalized (3.10 vs. 0.53 and 0.42, respectively for wt and AL1 and AL3 KO; *n* = 3, *p* < 0.0005). Similar changes in NADP^+^ were not observed; furthermore, we observed, in general, more 100-fold lower NADP^+^ level in these three types of cells, and no significant difference between TS vs. ML or WT vs. KO cells (*n* = 3, *p* = 0.7). We expected also increased levels of the cytosolic folates. This was indeed the case. More whole cell folate isoforms such as formyl THF (0.001 vs. 0.010 and 0.009 µmol/g protein for wt vs. AL1 and AL3 KO, respectively *n* = 3, *p* < 0.007) and methyl THF (0.004 vs. 0.05 and 0.01 µmol/g protein for wt vs. AL1 and AL3 KO, *n* = 3, *p* < 0.0007) were detected along with increased levels of methionine, serine and glycine. The latter increases in 1C-bearing amino acids suggest that ALDH1L2 in cancer stem cells consumes an important amount of 1C units for the production of NADPH.

### 3.4. ALDH1L2 KO Cells Bear More ROS despite No Change in Glutathione Content

We subsequently determined the level of GSH and total glutathione as their changes can signal their involvement in NADPH-linked anti-oxidative response of the mitochondria. As shown in Figure 3II, no significant differences were detected between the three cell types in either TS or differentiated ML cells. As in normal cells, the redox homeostasis of the mitochondria is maintained mainly by glutathione, glutaredoxin, and thioredoxin systems, and we examined these systems in the U251 cell transcriptomes obtained from our previous study comparing the TS cells and the differentiated ML cells. No difference in the transcription of TXN, GSR and GPX family genes was found. No change was also found for another known redox buffer system peroxiredoxin III and its regulator sulfiredoxin [19]. The mitochondrial SOD2, however, was notably increased in the TS cells of U251, suggesting its involvement as the main antioxidative mechanism in TS cells of U251. In addition, we also examined the expression for the NOX genes since they may cause increased H_2_O_2_ and a free oxygen radical level. Their transcription levels were nondetectable.

The impact of reduced NADPH levels on overall ROS levels of the *ALDH1L2* KO TS cells were examined as ROS has been established as a negative regulator for stemness genes in both normal as well as cancer stem cells including glioblastoma stem-like cells [20,21]. We adapted a commercially developed non-fluorescence cell permeable ROS detection dye broadly used to detect the ROS in live cells [22]. It reacts directly with wide varieties of reactive species like hydrogen peroxide, peroxynitrite and hydroxyl radicals, and resulting in products emitting green fluorescence capable of detection by microscopy to indicate the cellular contents of ROS/RNS (see Supplemental Methods). As shown in Figure 3II, the ROS level in wild type is significantly less than that observed in both AL1 and AL3 knockout cells in both ML and TS states (9.15 vs. 26.97 and 14.91 for ML cells (*n* = 5 vs. 6 and 5 in wt vs. AL1 and AL3 KO, respectively; *p* < 0.004) and 8.61 vs. 22.82 and 21.50 for TS cells (*n* = 8 vs. 13 and 6 in wt vs. AL1 and AL3, respectively; *p* < 0.007).

### 3.5. Mitochondria Morphological Studies

Previous evidence has suggested that, for non-neoplastic stem cells, there is a close connection between stemness and mitochondrial morphology and function, with fewer mitochondria and less developed cristae (hence, less OXPHOS metabolism) associated with less differentiated cells [23]. In cancer stem cells, although mitochondria dysfunction is linked to the so-called hallmarks of cancer [24,25], no fixed metabolic phenotype is associated with cancer stems cells. For example, cancer stem cells of the glioblastoma [26] along with lung cancer [27] and pancreatic ductal adenocarcinoma [28] have been found to exhibit OXPHOS metabolic phenotype while breast cancer [29], colon cancer [30], and glioblastoma U87 cells [31] exhibit glycolytic phenotype. Tumor tissue types and tumor microenvironment may thus all contribute to the metabolic plasticity of the cancer stem cells.

**Figure 4:**
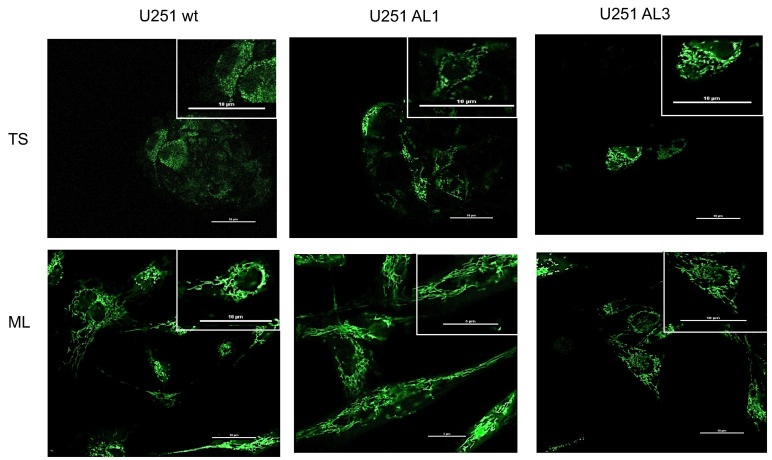
*ALDH1L2* knockout alters mitochondrial morphology. *ALDH1L2* knockout led to a visible change to the morphology of the mitochoindria of the KO TS cells. Relative to control wt TS cells, the mitochondria of the KO TS cells have a larger network-like structure, where, in wide type TS cells, the mitochochondria are much more fragmented with an oval-like shape. Mitochondria in U251 tumor spheres and in adherent U251 cells were labeled with green fluroescent protein (GFP) fused to the leader sequence of E1 alpha pyruvate dehydrogenase and observed under a confocal microscope (see Supplemental Methods).

Here, to know the metabolic phenotype of the U251 cells and the effect of knocking out *ALDH1L2*, we compared the morphology of the mitochondria of the wild type vs. the knockout cells. As shown in Figure 4, in wild type U251 cells, the mitochondria of the TS cells is much more fragmented than those of the ML cells. This difference between mitochondria network of the TS vs. ML cells appears to diminish with the knockout of the *ALDH1L2*. In knockout TS cells, the organization of the mitochondria network looks more like that of the ML cells. Besides this difference, we noted that the TS of the knockout cells exhibits a much greater tendency to differentiate into ML cells when the TS was placed on poly-lysine coated cover glass (data not shown). Within less than 30 min, the TS of the knockout flattened out onto the cover slide, with a fully developed mitochondria network identical to that of the ML cells.

## 4. Conclusions

One key feature of cancer cells is metabolic reprogrammation. The molecular details of the reprogramming of 1C metabolism in cancer are not well known. Even fewer studies have been published on the mechanisms by which altered 1C metabolism may influence the fate of cancer stem cell (CSC). Using a 3D in vitro culture technique [32], we studied the CSC of the glioblastoma in the form of tumorospheres (TS), and found a profound alteration in 1C metabolism at the level of a mitochondria folate cycle [18]. This earlier work revealed that mitochondria folate enzyme *ALDH1L2* is much more expressed in TS than in differentiated glioblastoma cells; in addition, the growth of the TS cells is much more methionine dependent than that of the differentiated cells. To understand these differences, we created the knockout of *ALDH1L2*. Here, we report the creation and the consequences of the *ALDH1L2* knockout in U251 cells.

ALDH1L2 protein is a 1C unit spender. This mitochondria folate-dependent aldehyde dehydrogenase takes the 1C from the formyl group of its immediate substrate 10-formylTHF and converts it into CO_2_. As this conversion requires NADP as an electron acceptor, it produces NADPH as a consequence. A knockout of this enzyme may thus abolish the methionine-dependence of the TS growth. Our data here showed that this is indeed the case. Such reprogramming of 1C metabolism in wild type TS cells is expected to improve their reducing power for anabolic reactions and redox balance, albeit it can end up with a reduced cellular proliferation if exogenous methionine is not provided. The carbon wasting of ALDH1L2 thus explains the dependence of TS cells on methionine.

Besides the normalized methionine dependence, the TS of the *ALDH1L2* knockouts exhibit two other features suggestive of the possibility that these KO TS cells are less stem-like than the wild type TS cells. First, the morphological difference between mitochondria of the wt vs. that of the KO TS is indicative of the fact that the metabolic phenotype of the former may be less OXPHOS (oxidative phosphorylation) than that of the latter, as the former shows a far-more extensive mitochondrial network. In fact, when placing the tumor spheres onto cover glasses coated with polylysine for observation under a microscope, the KO TS quickly attached onto the cover glass, and flattened into adherent monolayer-like cells with an extensive mitochondria network; no similar transformation was observed for wt TS. Second, consistent with the morphological finding, using Oxygraphy, we found the metabolic phenotype of the knockout tumorospheres tend to be more OXPHOS as the Bioenergetic Health Index [33] is significantly higher in KO vs. wild type cells (data not-shown).

In conclusion, our evidence reported here supports the notion that the upregulation of ALDH1L2 is key to the formation of cancer stem cells of the U251 glioblastoma cells, and its function appears to be related to the production of NADPH via the utilization of 1C-units delivered by mitochondria 10-formyl THF. As this conversion consumes 1C-units, it reduces the level of mitochondria formate, and hence the level of cytosolic formate, the 1C-unit donor needed for cytosolic methionine synthesis. This leads to the methionine dependence of the CSC formation reported in our earlier work [18]. We also suggest that the increase in NADPH production generated by the upregulation of ALDH1L2 is used to reduce the ROS level, a condition needed to maintain the stemness characteristic of the cancer stem cells.

## Data Availability

All data are available upon request.

## Supplementary Materials

The Supplementary Materials information can be downloaded at: https://www.mdpi.com/article/10.3390/nu14091887/s1.
