# Supplementary material for: ALDH1L2 Knockout in U251 Glioblastoma Cells Reduces Tumor Sphere Formation by Increasing Oxidative Stress and Suppressing Methionine Dependency
Source: Nutrients. 2022 Apr 30;14(9):1887. doi: 10.3390/nu14091887 (PMC9105572; doi:10.3390/nu14091887)
Supplement: Supplementary file 1 [file nutrients-14-01887-s001.zip › nutrients-1588160-supplementary.pdf]

Supplemental Methods

Cell Culture

The U251 cells used were authenticated by ECACC (European Collection of Authenticated Cell Cultures) and purchased from Sigma Aldrich (09063001, France). The HEK293 cells were from ATCC (CRL-11268, USA). For the study of cancer stem-like cells in glioblastoma, 2 culture conditions were used with U251 cells: adherent 2D monolayer (ML) and non- adherent 3D culture in the form of tumor spheres (TS) at 37°C under 5% CO2 (PMID: 31395852). Briefly, the adherent cells were grown in standard culture dishes with DMEM-F12 (D6434 Sigma-Aldrich) supplemented with 10% Fetal Calf Serum (FCS) (F7524 Sigma-Aldrich) and 100mg/mL penicillin-streptomycin (P4333 Sigma-Aldrich), and the TS cells in non-adherent 6-well plates pre-coated with 2% 2-hydroxyethylmethacrylate (P3932 Sigma-Aldrich) at a density of 20,000 cells/well with DMEM-F12 supplemented with 1XB27, 0.2 µg/ml EGF,0.2 µg/ml FGF (17504044, PHG0311, PHG0026 Fisher Scientific, France) and 100mg/mL penicillin-streptomycin. Only passage 3 of the TS with diameter > 50µm were used for the study. Their quantification was achieved by using MoticamX (Motic Europe S.L.U., Barcelona, Spain) and Image J software. In experiments where various methionine concentrations were included in culture media, methionine free DMEM (21013024, Fisher Scientific) was used, and it was supplemented various amount of methionine (M2768 Sigma-Aldrich), L-cysteine at 30 mg/L (C7352 Sigma-Aldrich), penicillin/streptomycin 1%, and B27/FGF/EGF for TS or 10% FCS for ML. All cells were routinely screened to control possible mycoplasma contamination (Venor GEM one step, MB Minerva Biolabs, Germany).

Hek293 cells were used to produce lentiviral particles. They were cultured in DMEM high glucose (11965 Thermo Fisher) supplemented with heat inactivated 10% Fetal Calf Serum (FCS) (10082147

Thermo-Fisher) and 100mg/mL penicillin-streptomycin at 37°C and under 5% CO_2. Twenty-four hours prior to transfection, HEK293 cells were seeded at a density of $1{,}5X10^6$ in T75 flasks with medium without antibiotics. These cells were then transfected according the protocol described below.

Lentiviral Construction

Plasmid lentiCRISPRv2 puro obtained from Addgene (98290, USA) was amplified and purified before used. After digestion and dephosphorylation with Esp3I (FastDigest BsmBI/Esp3I ER0451 New England Biolabs, USA), the linearized plasmid was purified (QIAquick Gel Extraction Kit (Qiagen, Germany). The single RNA guides targeting exon 1 (5′**CACCG**GCGCTCCGGCGCTTCTCCAC3′, 5′**AAAC**GTGGAGAAGCGCCGGAGCGCC3′) and exon 3 of human ALDH1L2 gene (5′**CACCG**GCAGAAGCCTACAGATCCGT3′, 5′**AAAC**ACGGATCTGTAGGCTTCTGCC3′) were separately phosphorylated, and annealed prior to the ligation with the linearized lentiCRISPRv2. The resulting lentiCRISPRv2-EX1 and lentiCRISPRv2-EX3 plasmid were then independently amplified in *Escherichia coli Stbl3*-competent bacteria (C737303 Life Technologies, Thermo Fisher Scientific, USA), and purified (#12941 Plasmid Plus QIAgen Midi prep, Qiagen). These RNA guide-inserted lentiCRISPRv2 plasmids were separately mixed with both pPAX2 (#12260 Addgene) and pCMV-VSV-G (#8454 Addgene) in OPTI-MEM Reduced Serum Medium (#31985070 Thermo-Fisher, USA) and then transfected into HEK293 cells using FuGENE 6 (Promega, USA) in Opti-MEM Reduced Serum Medium to produce lentivirus particles containing the guided CRISPR-Cas9 for exon 1 and exon 3 independently (protocol adapted from the Birtwistle Laboratory protocols: http://www.birtwistlelab.com/protocols). The titer of the lentiviral

preparations were then determined after concentration using lenti-X-concentrator (631232 Takara Bio Europe, France) according to the manufacturer's protocol.

Generation of ALDH1L2 CRSPR knockouts

To create ALDH1L2 knockout cells, U251 were transfected with the lentivirus particles using a polybrene (Hexadimethrine bromide, #H9268 Sigma) based protocol described by Addgene (https://www.addgene.org/protocols/generating-stable-cell-lines/). Forty-eight hours after transfection, these cells were treated with puromycine at 8µg/µL for 72h to select for transfected cells. This step was followed by sequential cloning using cloning rings. For each isolated clone, ALDH1L2 gene deletion was evaluated by targeted sanger sequencing. In brief, DNA from wild type and cloned knockout cells were extracted, and the CRISPR targeted exon 1 and exon 3 were amplified using PlatiniumTaq DNA polymerase (#10966034 Invitrogen) with the following primers for exon1 and exon, respectively: 5'AGGTTGGCTGTCCGAACG3'(F), 5'ACGCTGTCTTGGTGTTTAGC3'(R), and 5'TGGCTGCAGAGAAAGATGG3'(F), 5'GCCGTGCTTTGGACTATCA3'(R). The cleaned PCR products were cloned into pCR4-TOPO vector (TOPO TA cloning system K4575J10, Thermo Fisher) for transformation and selection in TOP 10 *E. coli* bacteria (C404010 Thermo-Fisher) with ampicillin. For each exon, at least 10 colonies were picked for amplification. Their plasmid DNA was subsequently isolated (QIAprep Spin Miniprep Kit 27104, Qiagen) and Sanger sequenced with the same primers used for PCR amplification mentioned above. Besides Sanger sequencing, ALDH1L2 protein expressions in wild type and knockout cells were also evaluated by capillary electrophoresis immunoassay.

Capillary electrophoresis immunoassay

Capillary electrophoresis immunoassay (Simple Western System Wes; ProteinSimple, USA) was used for the semi-quantitative comparisons of the protein of interested reported here. All materials except the primary antibodies used were obtained from ProteinSimple following the recommended protocols of the manufacture. For all experiments, cellular proteins were extracted with RIPA buffer and freezing/thawing cycles and aliquots of protein at 0,2μg/μL were prepared for a loading of 0,6 ug of protein for the electrophoresis. The antibodies used were anti-ALDH1L2 polyclonal antibody (at 1:100, NBP1-81935, Novus Biologicals, United Kingdom), and anti-vinculin antibody (at 1:500, 13901S Cell-Signaling, USA). The data were analyzed using Compass software (ProteinSimple).

Whole genome sequencing (WGS)

Whole genome sequencing (WGS) and primary analysis of the results were performed by the company BGI (BGI Tech Solutions HongKong Co. Limited 1 / F 16th Dai Fu Street Tai Po Industrial Estate Tai Po HongKong) on a BGISEQ500 sequencer. The secondary and tertiary analyzes (alignment of reads on the reference human genomic sequence, detection_annotation_visualization of variants) were carried out with the help of Prof. Franck Broly (Department of Genopathies, Center for Biology, Pathology and Genetics, Regional and University Hospital Center of Lille, France). The data were analyzed from Fastq BAM or VCF files generated by the BGI company with several software and in particular: Variant Studio (Illumina Inc: Worldwide Headquarters 5200 Illumina Way San Diego CA 92122 USA), Genesearch NGS (PhenoSystems SA Sentier des Communs 1807 Blonay Switzerland) Integrated Genome Viewer (IGV: Robinson JT et al Integrative Genomic Viewer Nature Biotechnology 2011, 29: 24-26) available free of charge at http://software.broadinstitute.org/software/igv/, HGVD pro (Human Genetic Variation Database http://www.hgvd.genome.med.kyoto-u.ac.jp), Varsome (The Human Genomic Variant Search

Engine (http://varsome.com) and Alamut (http: // www.interactive-biosoftware.com/fr/alamut-visual/)

High-performance liquid chromatography (HPLC) analysis

The levels of NADPH and GSH were analyzed using high-performance liquid chromatography (HPLC). For total cellular NADPH determination, the separation principle adapted here is based on the work of Lowry et al. (PMID: 14466980) who observed that while at elevated temperature $NADP^+$ is highly instable in alkaline solution, NADPH is highly instable in acidic solution. We thus heat-treated fresh cell or tumor sphere lysates under alkaline condition to assay the NADPH contents. Briefly, fresh cell/TS pellets were obtained by centrifugation (300 rpm, 5 min at room temperature) after washing them twice with 1X PBS. Aliquots of Cell/TS lysates were then prepared by adding 0.1X PBS to cell/TS pellets containing about 1-2 millions of cells to reach a final volume of 250 ul. The fresh cell/TS lysate was then alkalized by adding 2.5 µL of 2M NaOH to 50 µL of cell/TS lysate; this step was immediately followed by a 15-minutes incubation at 80°C to degrade $NADP^+$. For NADPH detection, the NADPH content of the heated alkaline lysates was further derivatized in the presence chloroacetaldehyde under acidic pH by adding 10µL of 3M acetate, at 80°C for 20 min (Katayama et al. PMID: 11522058) for end product detection by fluorescence detector after the HPLC separation outlined below. The product mixtures after derivatization reaction were centrifuged at 12,000g for 10 min at 4°C; 10 µL aliquots were injected into HPLC (Ultimate 3000, Thermo Dionex) equipped with a C18 Gemini column (Gemini H19-100671 / 5526-0064,150x4.6 mm, 3 µm, 40°C). The eluent gradient applied was a mixture of Solvent A (50 mM KH2PO4, 1% Methanol and 10 mM TBAOH pH 6) and B (50 mM KH2PO4 40% Acetonitrile pH 6), with a 0.8 mL/min flow rate. The eluates were monitored

with a spectrofluorometer (RF10AXL, Shimadzu) equipped with an excitation light set at 278nm, and emission at 418nm.

For total cellular glutathione determination, cell/TS lysates were prepared as those for the measurement of NADPH described above using 0.1X PBS. For cellular total reduced glutathione GSH, 5 µl aliquots of DTT at 10mM were first added to 40ul aliquots of cell/TS lysates for 10 minutes at room temperature. Aliquots of 100ul of ice-cold methanol (previously frozen at -20°C) were then added to the cell lysates to precipitate the protein contents (4°C, 30 minutes), and later removed by centrifugation at 12,000 g at 4°C. Speed Vac was then applied to evaporate the entire aqueous phase of the supernatants during 1 hour and 30minutes. This was followed by adding aliquots of the following solutions: first 45µL H20, then 5µL Tris-HCl 1M (pH 7.4), and finally 50 µL DTNB (5,5'-dithio-bis(2-nitrobenzoate)) 4 mM, with agitation during each step to completely resuspend the residue lysates. Further incubation of the lysates was performed in a mixing block at 37°C for 5 minutes at a rotational speed of 400 turns/min. Aliquots of 15 µL were injected into HPLC (Ultimate 3000, Thermo Dionex) for separation in Spherisorb ODS with a 0.8 mL/min flow rate. The eluent gradient was a mixture between Solvent A (NaH2PO4 (2 H2O) 50 mM, TBA-OH 5 mM, Acetonitrile 0.1%, pH 2.5) and B (NaH2PO4 (2 H2O) 50 mM, TBA-OH 5 mM, Acetonitrile 40%, pH 2.5) and eluates were monitored with a UV detector set to 335nm.

Both NADPH and GSH concentration (nmol/L) were normalized to total protein concentrations (g/L) of the lysates and results were expressed as nmol/g protein. Measurements were performed with a minimum of 3 independent cell cultures.

Metabolites analysis

Metabolites analysis including Methionine, Homocysteine, SAM, SAH, total Cysteine, Cystathionine, total Glutathione, Choline, Betaine, Glycine, Serine, methyl-THF, formyl-THF were realized by LCMS (LCMS 8045, Shimadzu, Kyoto, Japan) on a Kinetex column (Kinetex 00D-4462-EO). These measurements were performed on ML and TS cell lysates prepared as follows. Cell/TS pellets containing ~1-2 million cells were suspended first in 500µL of 0.1x PBS; to this suspension, a 250µL aliquot of DTT(100mM)-containing ascorbic acid solution (25mM) was added before breaking the cell membranes sequentially with 26G needle (15 cycles), sonication (30 minutes, 4°C), and thermic choc. Samples were then centrifuge 20,000g (30 minutes, 4°C); to precipitate the protein contents, the resulting supernatants were added with 10µL of 600mM DTT/formateNH4, 60µL of methanol/formic acid 0,1%, and incubated for 30 minutes at 4°C prior to centrifugation (20,000 rpm, 30 minutes, 4°C). Aliquots of 25µL of samples were loaded onto plates with 100µL of H_2O/0,2% formic acid and inserted into the LCMS/MS for injection. The eluent gradient was a mixture of solvent A (H_2O 0,1% TFA) and B (Acetonitrile, 0,1% formic acid). Metabolite concentration (nmol/L) were normalized to total protein concentrations (g/L) and results were expressed as nmol/g protein. Measurements were performed with 3 independent cell cultures.

Immunofluorescence assay

To observe mitochondria in live cells, we used CellLight™ Mitochondria-GFP, BacMam 2.0 (C10600, Invitrogen, France). For adherent cells, one day before observation they were seeded onto cleaned (rinsed 3 times with PBS 1X) microscope cover glasses (pretreated with poly-L-lysine for 1 hour at 37°C) in 4-well plates (5000 cells/ 500ul medium per well containing 3ul CellLight™ Mitochondria-GFP). For tumor spheres (TS) cells, passage 3 tu-

mor spheres were placed in 1ml TS medium in 6 well methacrylate coated plates containing 5ul CellLight™ Mitochondria-GFP. Both were protected from light during an incubation period (37°C, 5% CO_2) of 16 hours following manufacturer's instruction. After incubation, ML cells were directly observed under confocal microscope while TS cells were centrifuged at 300g for 5 minutes and placed onto polylysine coated cover glasses for an hour for attachment onto the cover glass for observation. Laser 488 nm line and 510nm bandpass were used for the observation of CellLight™ Mitochondria-GFP. The quantification of the fluorescence was performed using ImageJ software.

Intracellular ROS levels were evaluated using the Total ROS Detection Kit (ENZ-51011 ENZO Life Sciences, Farmingdale, NY, USA). Similar to that described above for CellLight™ Mitochondria-GFP observation. Both ML cells and TS were plated onto polylysine coated cover glasses prior to observation. The duration for polylysine attachment for ML cells was 24 hours, while for TS was one hour. Staining procedures used were identical for both cell condition, and was 20 minutes according manufacturer's instruction. Confocal setting used was identical to that described above for CellLight™ Mitochondria-GFP.

Immunohistochemistry

Adherent cells were cultured on p100 culture plates until they reached 75% confluence. They were then washed (1XPBS) and pelleted. Passage 3 tumor spheres were collected, washed, and pelleted. These pellets were then fixed with 4% paraformaldehyde, embedded in paraffin block, then cut to obtain 3μm slices for immunohistochemstry procedures carried out in Autostainer Plus (Agilent, USA). Briefly, slices were mounted onto Polysine® plates (LABONORD S.A., France). These plates were then dry for 5 minutes at 58°C, then at 56°C for another 1-2 hours. A hot bath PTLink DAKO

with citrate buffer pH 6.0 was used for antigen retrieval during 20 minutes. Antibodies used were diluted with EnVisionTM FLEX Antibody Diluent (Tris pH 7,2, NaN3 15nmol/L). Antibodies used included anti- CD133 (1/200, rabbit, ab19898, Abcam, UK), CD44 (60224-1-Ig Proteintech, USA), ALDH1L2 (NBP1-81935 Novus Biologicals, USA).

Supplemental Figure S1

S1 a and b. Mapping of the sequence reads at on-target site in ALDH1L2 exon 1 (a) and exon 3 (b). All reads spanning the cleavage sites of the CRISPR/CAS9 system used contain indels reinforcing our belief that ALDH1L2 gene is completely invalidated

S1 c and d. Nucleotide variations disrupting protein function in a subset of genes other than ALDH1L2 occuring in U251 ALDH1L2 exon 1 KO (c) and U251 ALDH1L2 exon 3 KO (d). Inspection of the reads alignment in the invalidated and wild type cells revealed that these variations are located in regions of poor sequencing quality responsible for a disorderly alignemnt caused by an inaccurate base calling and not an “off-target” site.

Supplementary Figure S1

a

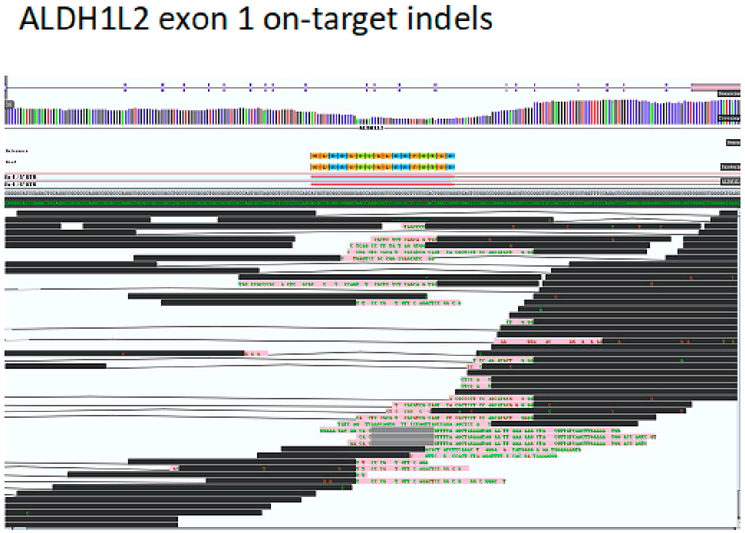

b

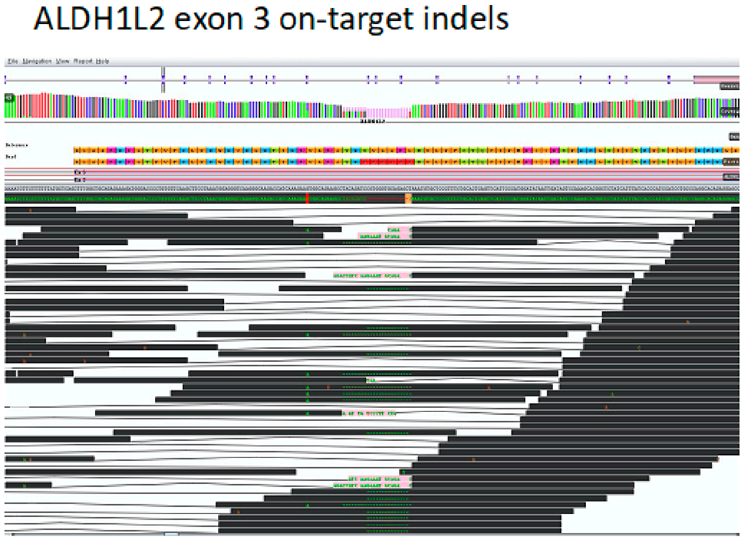

c

Chr.	Pos.	Name	AA626 U251 HD31	AA627 ALD1 HD31	Genes	Type	Known	Prediction	MAF
2	37384049	A>C		33,3%	EIF2AK2	Essential splice site	No		
4	47680072	C>A		31,4%	CORIN	Essential splice site	No		
9	140063306	T>C		29,7%	LRRC26 RP11-350O14.18 *GRIN1 TMEM210 MIR3621*	Stop lost	No		
5	76506751	A>G		31,8%	PDE8B	Start lost	No		
1	145014186	insA		34,4%	PDE4DIP *RP11-326G21.1*	Frameshift	No		
9	46390773	delGGAT		32,5%	FAM27D1 *FAM27E1 RP11-34H11.1 RP11-34H11.3*	Frameshift	No		
10	47192271	delG		33,3%	AGAP10 *RP11-144G6.4*	Frameshift	No		
10	48237081	delC		41,7%	AGAP9	Frameshift	No		
7	90894458	insCCC		35,3%	FZD1	Inframe insertion	No		

d

Chr.	Pos.	Name	AA626 U251 HD31	AA1431 ALD3 HD32	Genes	Type	Known	Prediction	MAF
4	47680072	C>A		39,5%	CORIN	Essential splice site	No		
2	128385993	G>T		48,3%	MYO7B *RP11-286H15.1*	Stop gained	No		
7	65112634	G>T		31,2%	AC104057.1 *INTS4L2*	Stop gained	No		
9	140063306	T>C		31%	LRRC26 RP11-350O14.18 *GRIN1 TMEM210 MIR3621*	Stop lost	No		
2	97907101	insA		35,1%	ANKRD36	Frameshift	No		
9	42380186	delTACT		31%	ANKRD20A2 *RNU6-1269P*	Frameshift	No		
9	107546689	delT		35,6%	ABCA1	Frameshift	No		
